# Increased vegetable consumption in Japan using an incentivized health communication campaign with a quiz

**DOI:** 10.1017/jns.2025.18

**Published:** 2025-04-02

**Authors:** Akira Kyan, Koryu Sato, Naoki Kondo

**Affiliations:** 1 Department of Social Epidemiology, Graduate School of Medicine and School of Public Health, Kyoto University, Kyoto, Japan; 2 Faculty of Medicine, University of the Ryukyus, Okinawa, Japan; 3 Faculty of Policy Management, Keio University, Kanagawa, Japan; 4 Department of Health and Social Behavior, School of Public Health, The University of Tokyo, Tokyo, Japan

**Keywords:** Dietary habits, Vegetable intake, Incentive-based programs, Health communication, Noncommunicable diseases, Public health, HCC, health communication campaign, SES, socio-economic status

## Abstract

Dietary habits, particularly vegetable consumption, play a crucial role in preventing noncommunicable diseases. However, despite international guidelines advocating daily vegetable intake, adherence remains low across many populations. As a result, more focused efforts to boost vegetable consumption at the population level are essential. This study aimed to assess the impact of a health communication campaign (HCC) in City A, which combined information dissemination and incentives to promote vegetable consumption. In 2021, a new app-based vegetable quiz was introduced as part of the ongoing campaign, which had been implemented since 2017. Participants earned 10 points per correct quiz answer, which could be redeemed for product certificates, with a maximum of 30 points. To evaluate the effectiveness of the quiz, we analysed vegetable intake data from 786 quiz users. A multiple regression analysis was conducted to consider factors such as sex, age, body mass index, pre-campaign points, prior vegetable intake, and frequency of food recording during the campaign. We ensured robustness of the results by analysing data from 605 individuals whose vegetable intake had been tracked one year earlier, during a non-incentivized version of the campaign. The results demonstrated that participants who completed all three quizzes consumed 10.7% more vegetables than non-participants. Year-over-year comparisons further showed a significant increase in vegetable intake among frequent quiz participants compared to the previous year, highlighting the positive impact of gamified quizzes on vegetable consumption. These findings suggest that incentivized HCC, especially those incorporating gamification elements, can be highly effective in encouraging healthier eating habits.

Noncommunicable diseases cause 41 million deaths annually, representing 74% of all deaths globally.^(1)^ Poor dietary habits, such as inadequate intake of omega-3 fatty acids, grains, and vegetables, are associated with increased risks of heart disease, certain cancers, type-2 diabetes, and obesity,^(2)^ and attributed to 16 million (1.0%) disability-adjusted life years and 1.7 million (2.8%) deaths worldwide.^(3)^ Vegetable intake has been demonstrated to increase the intake of a variety of nutrients and represent overall dietary quality; therefore, a promising strategy for ensuring adherence to dietary recommendations is endorsed.^(4)^ In this context, international guidelines advocate for at least three servings of vegetables daily (≥ 240 g/day).^(5)^ Nevertheless, survey data from 162 countries revealed that, by 2020, 88% of the population had insufficient vegetable intake.^(6)^ A 2018 audit of the World Cancer Research Fund International NOURISHING database, which catalogues global government nutrition policies and actions, reported 168 policies designed specifically to improve fruit and vegetable intake.^(7)^ However, a near-decade stagnation in policy approaches has been noted, focusing more on setting standards and raising public awareness rather than on actual changes in consumption,^(8)^ despite evidence that the dissemination of information is insufficient for impactful change.^(9)^ Thus, continued discussions and efforts are essential to increase vegetable consumption at the population level.^(2,10)^

A common measure for promoting general health at the population level is health communication campaigns (HCCs).^(11)^ HCCs apply integrated strategies to deliver messages designed directly or indirectly to inform, influence, and persuade targeted audiences about changing or maintaining healthy behaviours.^(12)^ Messages can be transmitted through various channels such as traditional mass media, the internet, social media, small media, group interactions, and one-on-one interactions.^(13)^ However, HCCs are not always promising, as a meta-analysis has shown that HCCs in general populations did not reduce mortality.^(14)^ Therefore, HCCs are sometimes implemented through incentives to enhance their effectiveness (e.g., the distribution of free or reduced-price health-related products). Such incentivized HCCs have been effective in improving health behaviours, such as smoking cessation and the use of child safety seats, condoms, and recreational safety helmets.^(15)^ The United States, France, and Australia have extended social prescriptions to promote vegetable consumption in socially vulnerable populations, providing vouchers, cash back, and free delivery of fruits and vegetables.^(16)^

In Japan, incentivized HCC cases have increased in prefectural and municipal governments since 2015. They offer incentives for favourable results of annual health checkups, health behaviours, such as the use of fitness gyms, dental checkup visits, recording the number of daily steps and diets, and participation in health programmes held by the local government.^(17)^ The incentives often take the form of “points” that can be exchanged for prizes or discount coupons at commercial facilities in the community. However, despite the attention paid to citywide incentivized HCCs, their effectiveness remains largely unexamined. Although one study demonstrated an association between HCC and physical activity,^(18)^ no studies have investigated incentivized HCC with a focus on vegetable intake.

Therefore, this study aimed to examine the association between citywide incentivized HCCs and vegetable consumption.

## Methods

### Setting and study participants

Our study was conducted in City A (anonymous by arrangement), a large Japanese city with a population of > 1.5 million. The population proportions of individuals aged < 19, 20–64, and > 65 years old in City A were 15.6%, 51.0%, and 27.5%, respectively. The demographic proportion was similar to that of representative large cities in Japan.

In 2019, City A began offering a smartphone application^(19)^ to implement incentivized HCC. HCC participants recorded their health behaviours such as vegetable consumption and daily steps recorded in the application and earned redeemable points according to their healthy behaviours.^(20)^ The application was provided free of charge and was available to all City-A residents.

Every September, City A conducted the “Let’s Eat Vegetables Campaign” (“*Yasai Wo Tabeyou Campaign*” in Japanese) to promote vegetable consumption among residents. During the campaign period, activities to promote vegetable intake—posters encouraging vegetable intake displayed at supermarkets and other stores in the city, distribution of recipe books using vegetables, and food-related events held at shopping centres and event venues in City A—were conducted.

In the 2021 campaign (from August 31, 2021, to September 30, 2021), participation in the campaign was promoted using the above-mentioned application. Specifically, a quiz on vegetables was distributed through the application thrice during this period (on August 31, September 14, and September 28), with 10 points awarded for each correct answer, resulting in up to a maximum of 30 points. The quiz content is provided in Appendix Material 1. The application provided explanations for both correct and incorrect answers.

We analysed the app data, including the users’ demographic characteristics, height, weight, health-behaviour records, and number of points registered in the healthcare application to examine whether this incentivized vegetable quiz influenced vegetable intake among residents. Of the total 6,693 app downloads by the end of the campaign period, the analytical sample included 786 people with an app record of vegetable consumption during the campaign and one month before it. In addition, the result robustness was confirmed using data from 605 participants for whom information on vegetable intake had been registered at the same time one year earlier, i.e., during the campaign period without incentive.

### Measures

The primary outcome was the individual mean daily vegetable intake during the campaign period. Daily vegetable consumption was identified from dietary records registered in the application, which allowed users to log every meal, with artificial intelligence enhancing accuracy through photo recognition of meals.^(21)^ It featured a comprehensive database of approximately 150,000 food items, including fresh foods, ready-made meals, and branded products, as well as menu options from approximately 450 establishments.^(21,22)^ Vegetable intake was adjusted for energy intake per 1000 kcal using the density method. Vegetable consumption validity was recorded in the app as previously established.^(21)^ The previous study assessed the validity of dietary intake data estimated by a healthcare application by comparing it with reference dietary records meticulously measured by nutrition experts, utilising these records as an external standard. The findings indicated that the estimation error for nutrient intake using the healthcare application ranged from −25% to +13%. Based on these results, the healthcare application was concluded to have a relatively high level of accuracy in dietary assessment.

Primary exposure referred to the number of responses to the quiz during the campaign. We assumed that this would be an indicator of how much the quiz incentives motivated the participants; therefore, we did not consider whether they answered the quiz correctly.

As covariates, we obtained data on sex, age, body mass index (calculated from height and weight), number of points earned before the campaign, vegetable intake in the month preceding the campaign, and number of campaign days with food entries using application information.

### Statistical analysis

The mean, standard deviation, and frequency of the quiz were calculated as the distribution of subject attributes. Given the sufficiently large sample size, the coefficient can be asymptotically approximated by a normal distribution according to the central limit theorem^(23)^. Thus, the association between the number of responses to the quiz and vegetable intake was estimated using multiple regression analysis after adjusting for covariates. In addition, as a robustness check, we compared vegetable intake between September 2020 (when the quiz had not been administered) and September 2021 (the campaign of interest), based on the number of responses to the quiz. All analyses were performed using Stata 18.0 (StataCorp, College Station, TX, United States).

## Results

Table 1 shows the participants’ characteristics according to the number of quizzes they answered. Comparing the demographics of the participants by the number of times they answered the quizzes, more women than men or older participants answered all three quizzes. They also accumulated more points on the healthcare app and tended to consume more vegetables before the campaign.

**Table 1.** Participants characteristics by answered-quiz number (N=786)

	Answered-quiz number				P-value
	0	1	2	3	
Women, N (%)	134 (42.4)	75 (54.0)	72 (55.4)	118 (58.7)	0.001
Age, mean (SD)	55.9 (13.5)	55.7 (12.0)	58.0 (12.0)	59.6 (10.8)	0.004
≤ 25 kg/m^2^ body mass index, n (%)	82 (26.0)	32 (23.0)	23 (17.7)	49 (24.4)	0.31
Medium or more of the health points, n (%)	82 (26.0)	64 (46.0)	91 (70.0)	156 (77.6)	< 0.001
Daily vegetable intake one month before the campaign, g/1000 kcal (SD)	152.4 (90.4)	159.9 (81.1)	162.9 (70.0)	179.9 (87.7)	< 0.001

SD, standard deviation. P-values are for the chi-squared and F-statistics.

**Fig. 1.**
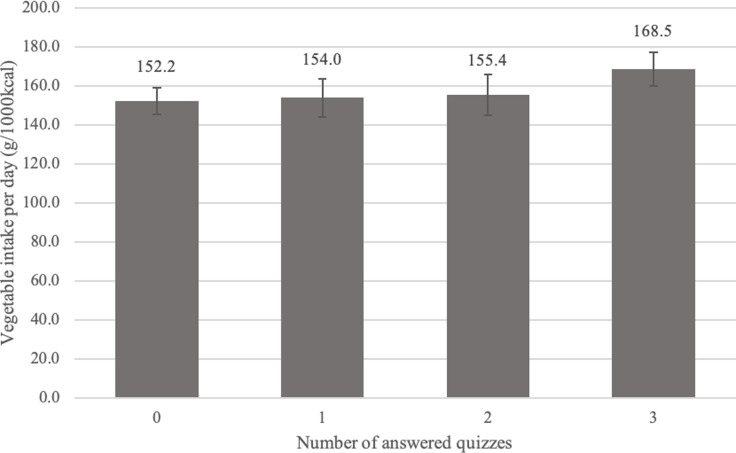
Association between number of quiz responses and vegetable intake (N=786). The error bars represent the 95% confidence interval. Adjusted for sex, age, body mass index, number of points, vegetable intake before the campaign period, and number of days dietary records were entered during the campaign period.

Figure 1 shows the results of the multiple regression analysis examining the relationship between the number of quiz responses and vegetable intake. Participants who answered all three quizzes had a mean daily vegetable intake of 16.3 g (95% confidence interval: 4.4, 28.2), i.e., 10.7% higher than that of those who never answered any quiz. However, no significant differences were found between non-responders and participants who answered the quiz once or twice.

**Fig. 2.**
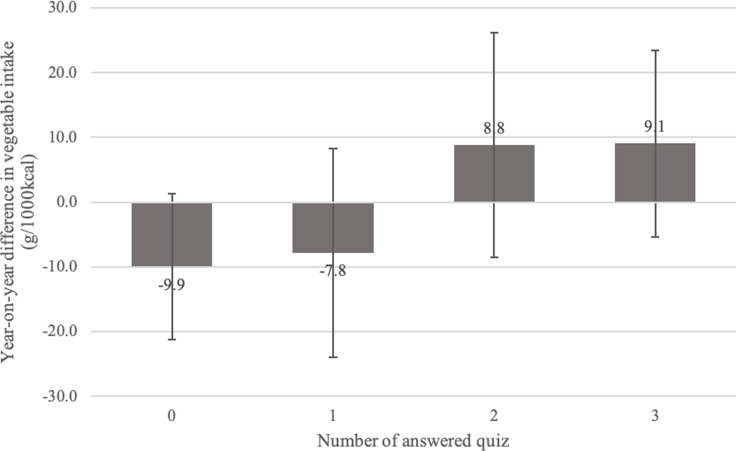
Association between number of quiz responses and year-to-year difference in vegetable intake (N=605). The error bars represent the 95% confidence interval. Adjusted for sex, age, body mass index, number of points, vegetable intake before the campaign period, and number of days dietary records were entered during the campaign period.

Figure 2 shows the results of the multiple regression analysis examining the association between the number of quiz responses and the year-to-year differences in vegetable intake. Participants who answered all three quizzes had a 19.0-g (95% confidence interval: −0.5, 38.4) greater year-to-year difference in vegetable intake than those who never responded to any quiz. Participants who answered the quiz less than twice decreased their vegetable intake by 2021 compared to by 2020, whereas those who answered the quiz twice or more tended to increase their vegetable intake by 2021.

## Discussion

This study examined the relationship between citywide HCC and vegetable consumption based on the number of responses to three incentivized vegetable quizzes, which provided information to promote knowledge and awareness and were administered through a healthcare application. Compared to non-responders, participants who answered all quizzes consumed more vegetables during the campaign. Furthermore, they tended to consume more vegetables than in the previous year, during which no quiz was administered.

Based on the baseline data, which indicate that individuals who answered the quiz multiple times may have had higher health consciousness and greater vegetable intake, participants who responded to all three quizzes may have had higher health consciousness and vegetable intake.^(24)^ Even after adjusting for covariates such as the number of points earned and the number of days dietary records were kept — both used as proxies for health consciousness — the results confirmed that vegetable intake during the campaign period was 10.7% higher among responders compared to non-responders. This finding suggests that the positive association between the quiz participation and vegetable intake may, at least in part, exist independently of health consciousness. We examined the changes in vegetable intake during the coronavirus disease 2019 outbreak using the same app and found a 6% increase in vegetable intake during the pandemic.^(22)^ Although a simple comparison cannot be made because of the different target populations, the 10.7% increase observed in the present study is notable compared to the previous study.

We also found that vegetable intake tended to increase among groups who answered the quiz two or more times in 2021 compared to their vegetable intake in 2020 when the quiz was not administered. Notably, vegetable intake increased despite no direct incentives for vegetables, such as vouchers and discounts. The quizzes included questions on vegetable intake standards, general vegetable deficiency in the Japanese population, nutrients contained in vegetables, and local products related to vegetables in City A. This information reinforced the participants’ knowledge and awareness regarding vegetable intake, which may have stimulated vegetable consumption.

This finding supports the conclusions of observational studies suggesting that digital interventions can effectively target behavioural change mediators, such as knowledge, attitudes, and cooking skills related to vegetable consumption.^(25,26)^ Given the concerns raised regarding the cost-effectiveness of app usage in HCC,^(9)^ gamification elements used in this study may present a viable strategy to enhance engagement and usability. Gamification is defined as “the use of game elements and mechanics in non-game contexts”.^(27)^ While financial incentives act as extrinsic motivation, gamification can facilitate intrinsic motivation by stimulating autonomy, competence, and relatedness.^(28)^ Thus, it is recognised as a powerful tool for improving health behaviours.^(29)^ Consequently, the quiz may have intrinsically motivated the participants by promoting enjoyment and contributing to increased vegetable consumption.

Despite the incentive structure allowing users to earn points simply by using the app to encourage engagement,^(9)^ the proportion of active users remained at approximately 10%. This suggests that the effects observed in this study may be limited to specific population groups. However, an Australian community intervention study evaluating the effectiveness of an app that provided nutritional information, suggested recipes, and offered tracking functions for vegetable intake and goal achievement reported that only 4% of users remained active after 90 days. In comparison, the proportion of active users in this HCC initiative is not relatively low. Although beyond the primary objective of this study, it is noteworthy that the number of users increased by approximately 30% in the year quizzes were introduced compared to the previous year.

Nonetheless, this study bore a few limitations. First, the generalizability of our findings may be limited because City A is a metropolitan area in Japan. Therefore, further studies are required to determine whether similar results can be obtained in suburban or rural areas, or in other countries or regions. Second, we could not obtain variables related to socio-economic status (SES). People with a high SES consume more vegetables than those with a low SES.^(30)^ It is also possible that this part of the population answered the quiz more actively. Therefore, omitting a potential confounder can lead to overestimation. However, we showed that the same individuals who actively answered the quiz tended to consume more vegetables in the year when the quiz was given than in the years when there was no quiz. Third, the quiz included several elements. It is impossible to determine whether the favourable results in this study were due to the content, difficulty, and acceptability of the quiz or the incentive provided. To elucidate the mechanism underlying the effect of quizzes on vegetable intake, it is necessary to investigate potential mediating effects by longitudinally collecting data on intermediate outcomes such as awareness and attitudes. Although cost-effectiveness should also be considered before social implementation, it was not considered in this study.

### Conclusions

Based on these results, individual vegetable intake can be increased via an incentivized knowledge awareness campaign using a health app which could increase commitment to HCC to promote vegetable intake. Although vegetable discounts and rebates have been shown to increase vegetable intake in vulnerable populations,^(16)^ to our knowledge, this is the first study to demonstrate the effect of promoting HCC participation through incentives for vegetable consumption in the general population. This study adds to the potential for effective population-approach strategies to provide information with incentives.

## Data Availability

Data are available upon reasonable request to City A.
